# Supplementary material for: Human Ubiquitin-Specific Peptidase 18 Is Regulated by microRNAs via the 3'Untranslated Region, A Sequence Duplicated in Long Intergenic Non-coding RNA Genes Residing in chr22q11.21
Source: Front Genet. 2021 Feb 3;11:627007. doi: 10.3389/fgene.2020.627007 (PMC7901961; doi:10.3389/fgene.2020.627007)
Supplement: Supplementary file 1 [file Data_Sheet_1.pdf]

Supplementary Information for:

Human USP18 is regulated by miRNAs *via* the 3'UTR, a sequence duplicated in lincRNA genes residing in chr22q11.21

Erminia Rubino[1,2], Melania Cruciani[1], Nicolas Tchitchek[2,3], Anna Le Tortorec[4], Antoine D. Rolland[4], Önay Veli[1], Leslie Vallet[1], Giulia Gaggi[1], Frédérique Michel[1], Nathalie Dejucq-Rainsford[4], Sandra Pellegrini[1*]

[1] Unit of Cytokine Signaling, Institut Pasteur, INSERM U1221, Paris, France
[2] École Doctorale Physiologie, Physiopathologie et Thérapeutique, ED394, Sorbonne Université, Paris, France
[3] i3 research unit, Hôpital Pitié-Salpêtrière-Sorbonne Université, Paris, France
[4] UMR_S1085, Irset (Institut de recherche en santé, environnement et travail), EHESP, Inserm, Univ Rennes, F-35000 Rennes, France.

*Corresponding author : Sandra Pellegrini
Email: sandra.pellegrini@pasteur.fr

This PDF file includes:

Supplementary Tables S1 and S2
Supplementary Figures S1 to S9
Supplementary Sequences

Supplementary Table S1. Copies of the sequences annotated as exon 1 of *FAM247A-D* and *linc-UR-B1*.

Coordinates [a]	Strand [b]	Identity % (score) [c]
FAM247A-D		
**chr22:18769207-18769785** [d]	**-**	100 (578/578)
**chr22:18349936-18350514**	+	100 (578/578)
**chr22:21203454-21204032**	**-**	99.9 (576/578)
**chr22:18511886-18512464**	+	99.9 (576/578)
chr20:23980580-23981153	**-**	96.2 (536/578)
chr13:18248901-18249486	**-**	96.6 (533/578)
chr22:22651789-22652345	+	96.6 (531/578)
chr22:24632871-24633427	+	96.6 (525/578)
chr22:24244569-24245122	**-**	96.6 (490/578)
linc-UR-B1		
**chr22:18861451-18861733**	+	100 (283/283)
chr22:21297396-21297678	+	100 (283/283)
chr22:21112840-21113122	**-**	99.7 (281/283)
chr6:162068502-162068656	**-**	82.6 (101/283)

[a] Blat was performed on human genome using *FAM247D* or *linc-UR-B1* annotated exon1 sequence as input.
[b] Refers to the genomic DNA strand
[c] Hits with identity > 80% and length > 100bp are shown.
[d] In bold coordinates referred to *FAM247A-D*.

Supplementary Table S2. Sequences of the primers used.

Primers	Sequences 5'-3'
USP18_qPCR_FW	ACTCCTTGATTTGCGTTGAC
USP18_qPCR_RV	TTTCCCACGGGTCTTCTT
OAS1_qPCR_FW	TTGACTGGCGGCTATAAACC
OAS1_qPCR_RV	TGGGCTGTGTTGAAATGTGT
IRF7_qPCR_FW	GGGTGTGTCTTCCCTGGATA
IRF7_qPCR_RV	GCTCCATAAGGAAGCACTCG
IFIT1_qPCR_FW	TCTCAGAGGAGCCTGGCTAA
IFIT1_qPCR_RV	TCAGGCATTTCATCGTCATC
STAT2_qPCR_FW	TATCACAGCCAGTGCCAGAG
STAT2_qPCR_RV	CTGATTCCCATCCTTGGAGA
18S_qPCR_FW	CATGGCCGTTCTTAGTTGGT
18S_qPCR_RV	CGCTGAGCCAGTCAGTGTAG
ACTB_qPCR_FW	TACAGCTTCACCACCACGG
ACTB_qPCR_RV	TGCTCGAAGTCCAGGGCGA
USP183UTR_XhoI_FW	CGCGCTCGAGTGGAAATGCCCAAAACCTTC
USP183UTR_NotI_RV	GCGCGCGGCCGCTCATGACTGTGTTTATCAC
191-5P_BSmut_FW	AAGACTCCGTAGATCCAGGATGCCTAATGGAAAATGACAGCGTGTCAATCTCTG
191-5P_BSmut_RV	CAGAGATTGACACGCTGTCATTTTCCATTAGGCATCCTGGATCTACGGAGTCTT
24-3P_BSmut_FW	GTTACATATTTTGATAATATCCCTAATTATAAATAAGCGAGTGTTATATAGTTTGAAAACAATGCTTCTCCTCATTGCA
24-3P_BSmut_RV	TGCAATGAGGAGAAGCATTGTTTTCAAACTATATAACACTCGCTTATTTATAATTAGGGATATTATCAAAATATGTAAC
423-5P_Bsmut_FW	TTATCAAAATATGTAACCATGAGGCGGGAGAGGTCCTGATCAGTCAGAATGGATG
423-5P_Bsmut_RV	CATCCATTCTGACTGATCAGGACCTCTCCCGCCTCATGGTTACATATTTTGATAA
532-3P_BSmut_FW	CCAGTGGGGAGAGCAGTGGCAGTCCCTCGCATCTGGGGGC
532-3P_BSmut_RV	GCCCCCCAGATGCGAGGGACTGCCACTGCTCTCCCCACTGG
3UTR_GSP1	AGTTGTATAATACTGAAG
A_FW1	CTGTTGCTGCTGACTCCAAG
A_RV1	TCCGTAGATCCAGGAACGGAA
A_FW2	TGAGGCATGAGTTTGGCCAC
B_FW1	TGGCCCAGGCAAGATAAATA
B_RV1	TGGTGAAAGCATCCATTCTG
B_FW2	CATTGATTACGACTTCCCTTCACCAC
B_FW3	CTTCTAACCCAGAGAACACAGC
B_FW4	CCTGGTGTCCATGCTTTTGTGA
B_FW5	CTCAGTTTCTGGTTACATCTGA
B_FW6	GCTGGCACTGCGAGCAATATA
3UTR_RV	TGAGGGGCCTCATGGTTACA

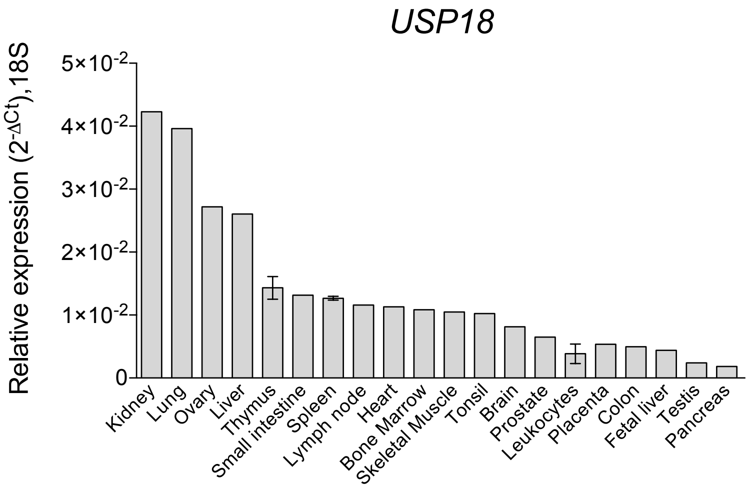

Supplementary Figure S1. *USP18* is widely expressed in human tissues.

Expression of *USP18* in 20 human tissues (Human Immune System MTC™ Panel, Human MTC™ Panel I, Human MTC™ Panel II). Results shown as expression ($2^{-\Delta Ct}$) relative to *ACTB*, used as housekeeping gene. SEM is shown for tissues that are present in Human Immune System MTC™ Panel and Human MTC™ Panel I.

A

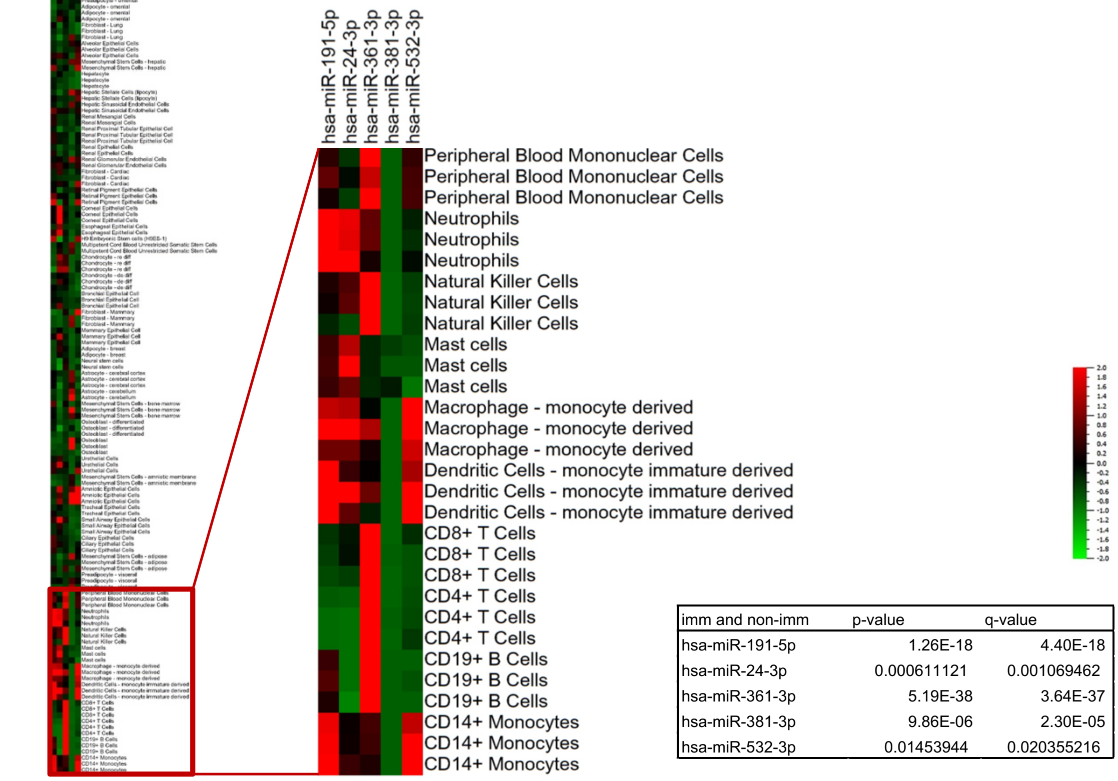

B

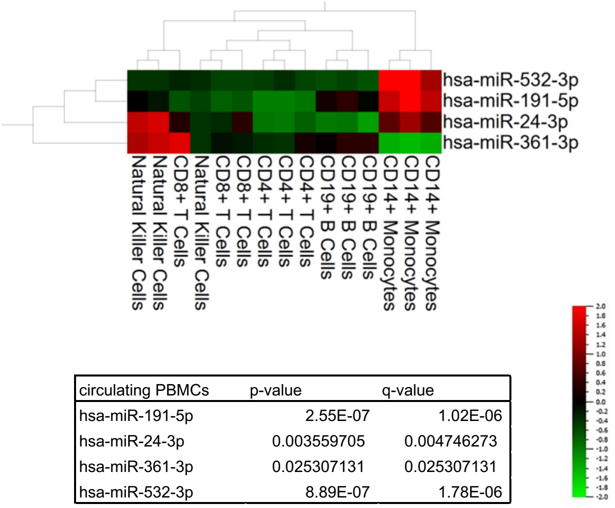

circulating PBMCs	p-value	q-value
hsa-miR-191-5p	2.55E-07	1.02E-06
hsa-miR-24-3p	0.003559705	0.004746273
hsa-miR-361-3p	0.025307131	0.025307131
hsa-miR-532-3p	8.89E-07	1.78E-06

imm and non-imm	p-value	q-value
hsa-miR-191-5p	1.26E-18	4.40E-18
hsa-miR-24-3p	0.000611121	0.001069462
hsa-miR-361-3p	5.19E-38	3.64E-37
hsa-miR-381-3p	9.86E-06	2.30E-05
hsa-miR-532-3p	0.01453944	0.020355216

Supplementary Figure S2. *miR-191-5p*, *miR-24-3p* and *miR-532-3p* are enriched in immune cells and particularly in monocytes.

(A) Heat map visualization of the data in Figure 2A. Differential expression of *miR-191-5p*, *miR-24-3p*, *miR-361-3p*, *miR-381-3p* and *miR-532-3p* in 90 human cell types (immune and non-immune) is shown. Immune cells are delimited by a red box and zoomed. p-values and q-values (ANOVA FDR adjusted p-value) are shown on the right of the heat map for all significant miRNAs.

(B) Heat map visualization of the data in Figure 2B. Differential expression of *miR-191-5p*, *miR-24-3p*, *miR-361-3p* and *miR-532-3p* in circulating PBMCs is shown. p-values and q-values (ANOVA FDR adjusted p-value) are shown below the heat map for all significant miRNAs.

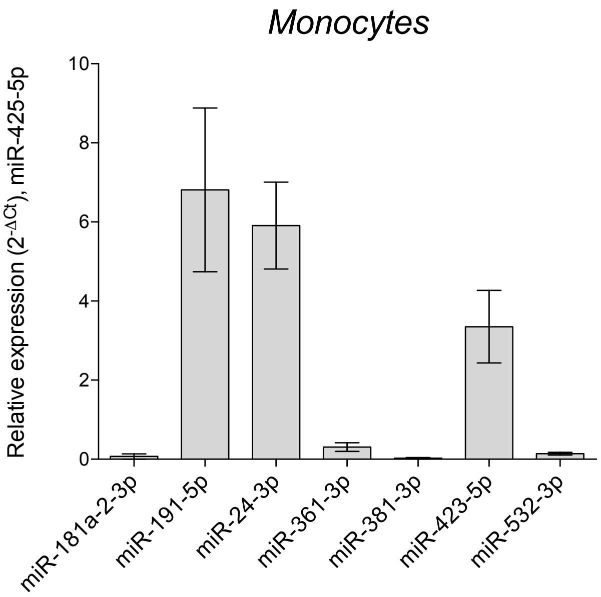

Supplementary Figure S3. ***USP18*****-targeting miRNAs in monocytes.**

The expression of the indicated *USP18*-targeting miRNAs in monocytes was measured by miRNA qPCR-array. *miR-425-5p* was used as normalizer

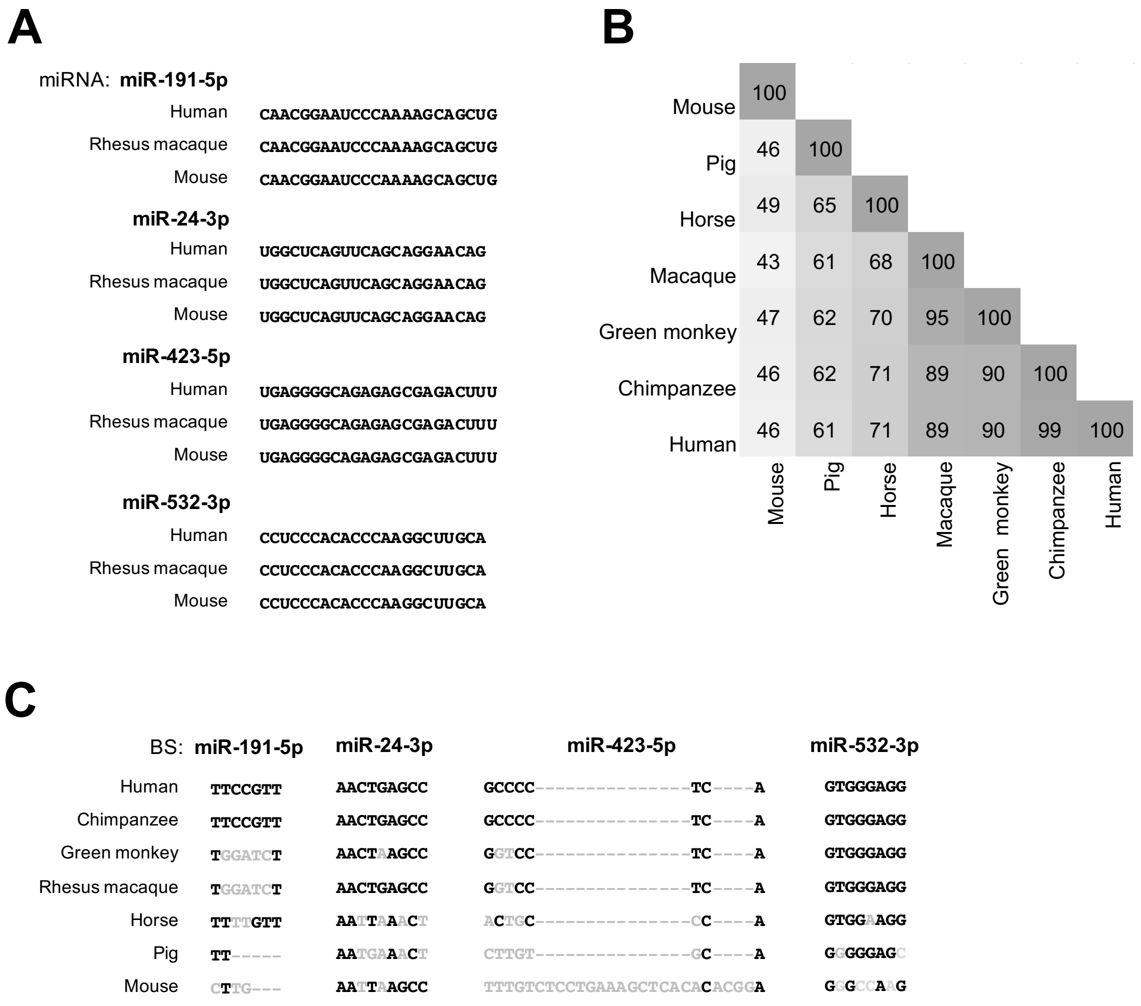

Supplementary Figure S4. Conservation of *USP18* 3'UTR and miRNA binding sites.

(A) Conservation of *miR-191-5p*, *miR-24-3p*, *miR-423-5p* and *miR-532-3p* mature sequence in rhesus macaque (non-human primate) and mouse. Conserved nucleotides in black. Sequences retrieved from mirbase (www.mirbase.org).

(B) Identity matrix of *USP18* 3'UTR in mammals. Each number indicates the percentage of identity between two species. Shades of gray indicate level of conservation (low in light gray; high in dark gray). *USP18* 3'UTR sequences were downloaded from UCSC (https://genome.ucsc.edu). Sequences were aligned using Clustal Omega (https://www.ebi.ac.uk/Tools/msa/clustalo).

(C) Conservation of the seed-matched region of the binding site (BS) of *miR-191-5p*, *miR-24-3p*, *miR-423-5p* and *miR-532-3p* on the *USP18* 3'UTRs of mammals. Conserved nucleotides in black; non-conserved nucleotides in gray.

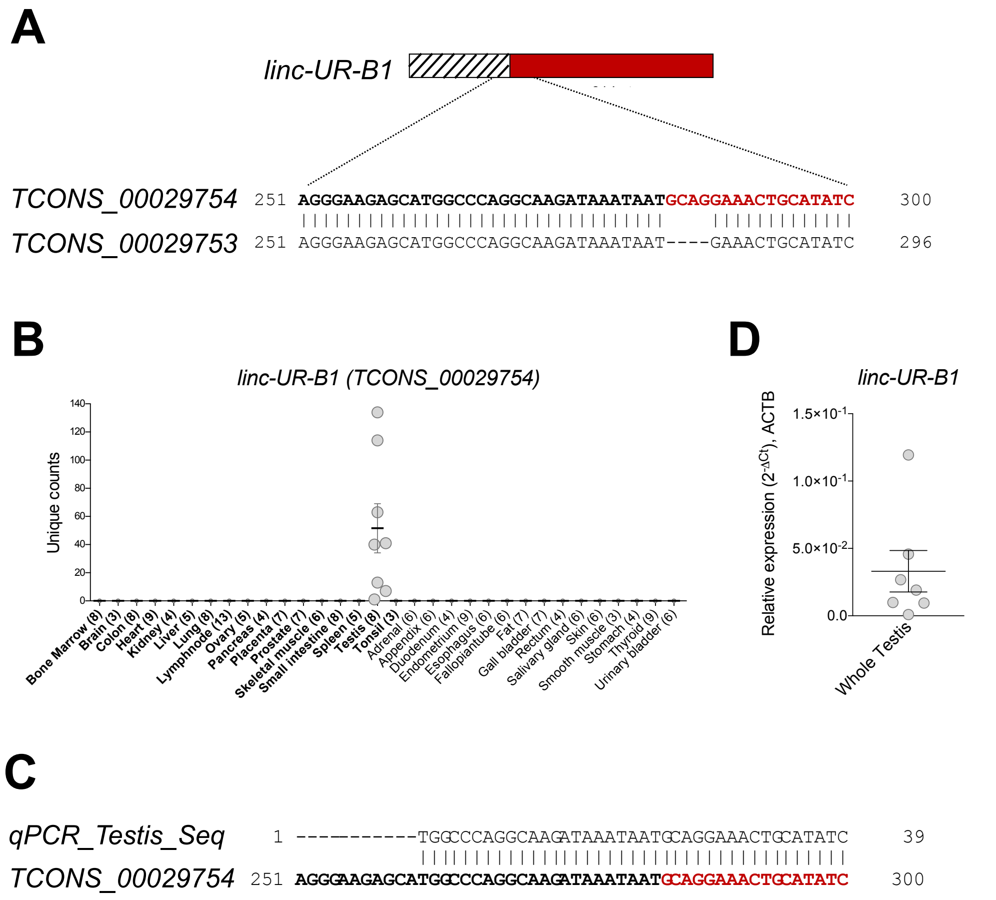

Supplementary Figure S5. *TCONS_00029754* is the *linc-UR-B1* isoform expressed in testis.

(A) Alignment of the exon-exon junction of *TCONS_00029754* and *TCONS_00029753* (putative *linc-UR-B1* isoforms). In the alignment, gaps are indicated with dashes. Note that an ATG codon in frame with the last 46 coding nucleotides of *USP18* is present only in *TCONS_00029754*.

(B) Expression of *TCONS_00029754*, here called *linc-UR-B1*, in 32 human tissues (RNA-seq data from https://www.ebi.ac.uk/gxa/experiments/E-MTAB-2836/Results), shown as unique counts. In bold the tissues in common with tissues in the panel analyzed by qPCR in Figure 4G. The other tissues are unique to this dataset. The number of donors is shown in brackets. *linc-UR-B1* detected only in testis (8 donors).

(C) Sequencing of the qPCR product obtained from testis cDNA in Figure 4G, revealed that the isoform of *linc-UR-B1* expressed in testis is *TCONS_00029754*.

(D) Expression of *linc-UR-B1* in testis fragments (7 donors), measured by qPCR. Expression is shown as relative ($2^{-\Delta Ct}$) to *ACTB*. The donor with the lowest expression of *linc-UR-B1* had impared spermatogenesis, while the other six donors

A

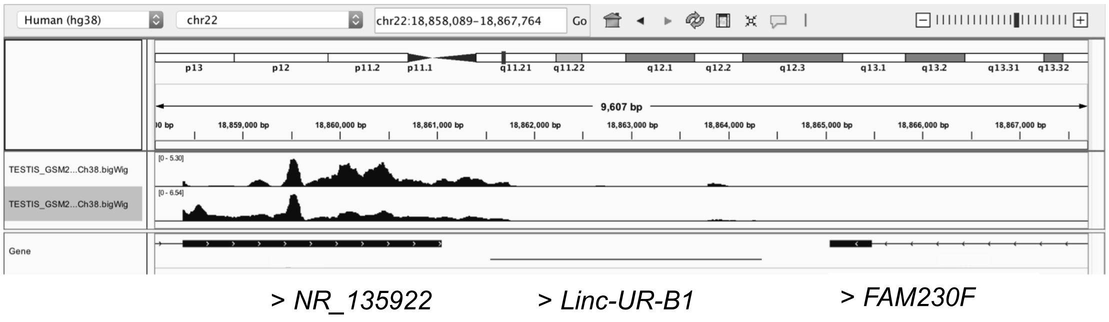

B

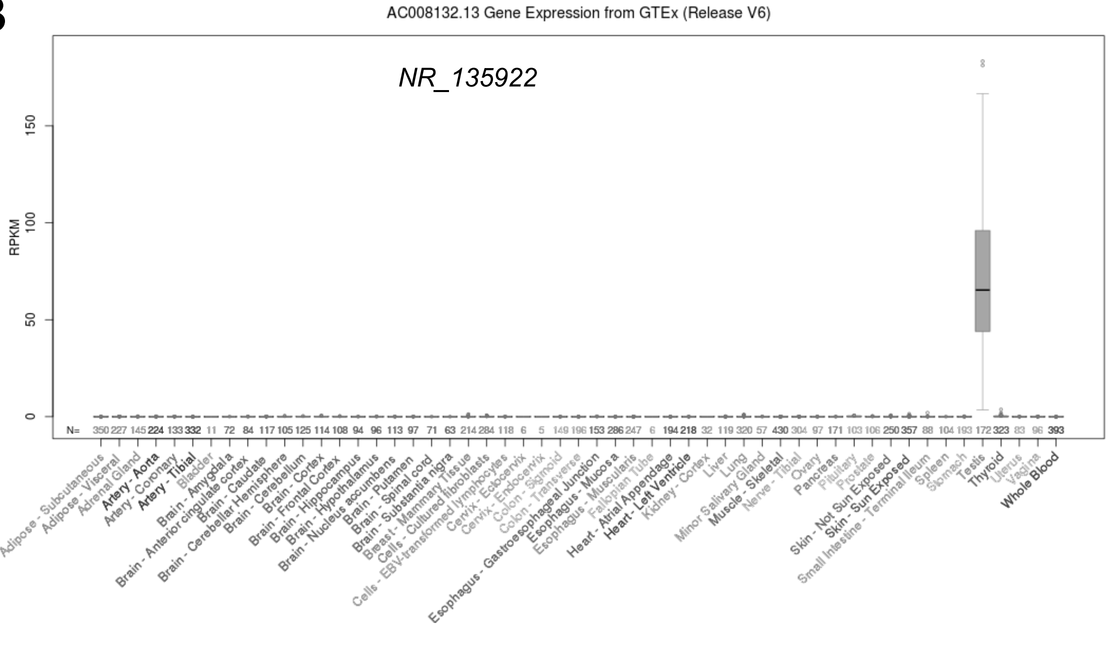

Supplementary Figure S6. The *NR_135922* pseudogene is uniquely expressed in testis. **(A)** PolyA+ RNA-seq (+ strand) track from testis provided by the ENCODE project (GSM2453457 and GSM2453458) were visualized using auto-scale mode in IGV browser (hg38 genome assembly). Genes are shown with their genomic orientation (> for + strand, < for – strand). *NR_135922* and the annotated intergenic region upstream of *linc-UR-B1* is covered by reads.

(B) Expression of *NR_135922* in GTEx data, retrieved from UCSC. *NR_135922* gene is under the name AC008132.12 and is expressed only in testis.

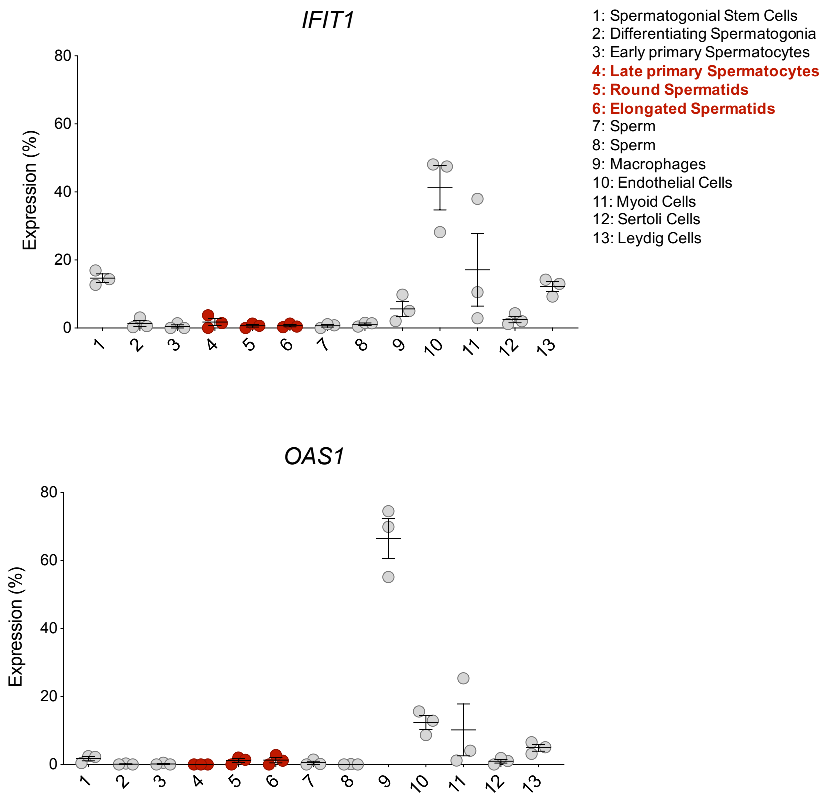

Supplementary Figure S7. No or low expression of ISGs in germ cells.

Analysis of *IFIT1* and *OAS1* in testicular cell populations (cluster 1-13, 3 donors). These data were retrieved from the alignment of single cell RNA-seq reads provided by (19), filtered for unique reads and expressed as percentage expression (normalized expression in one population *vs* all populations analyzed). Cell populations expressing *linc-UR-B1* are highlighted in red.

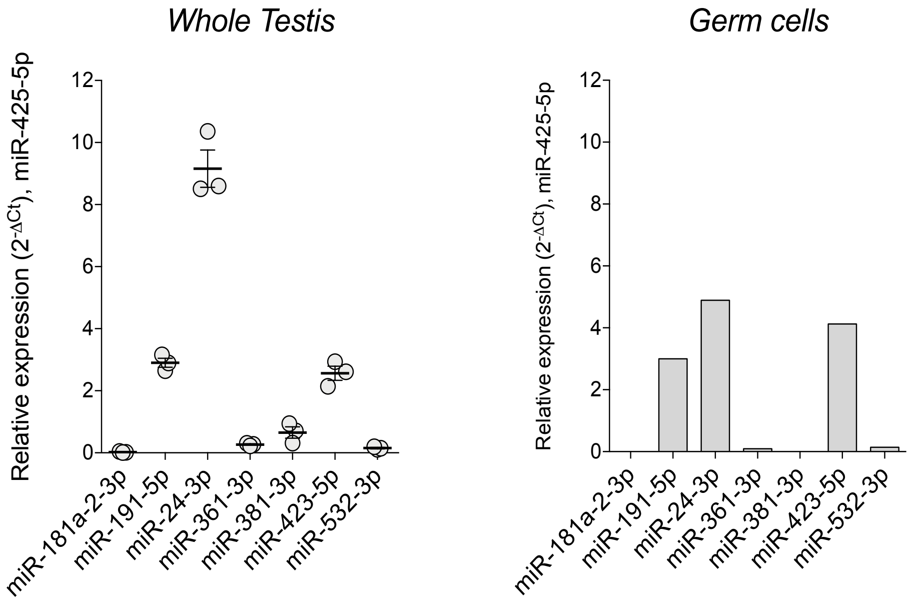

Supplementary Figure S8. *USP18*-targeting miRNA are expressed in whole testis and germ cells.

The expression of *USP18*-targeting miRNAs in testis fragments (left panel) and on purified germ cells (right panel) was measured by miRNA qPCR-array. *miR-425-5p* was used as normalizer.

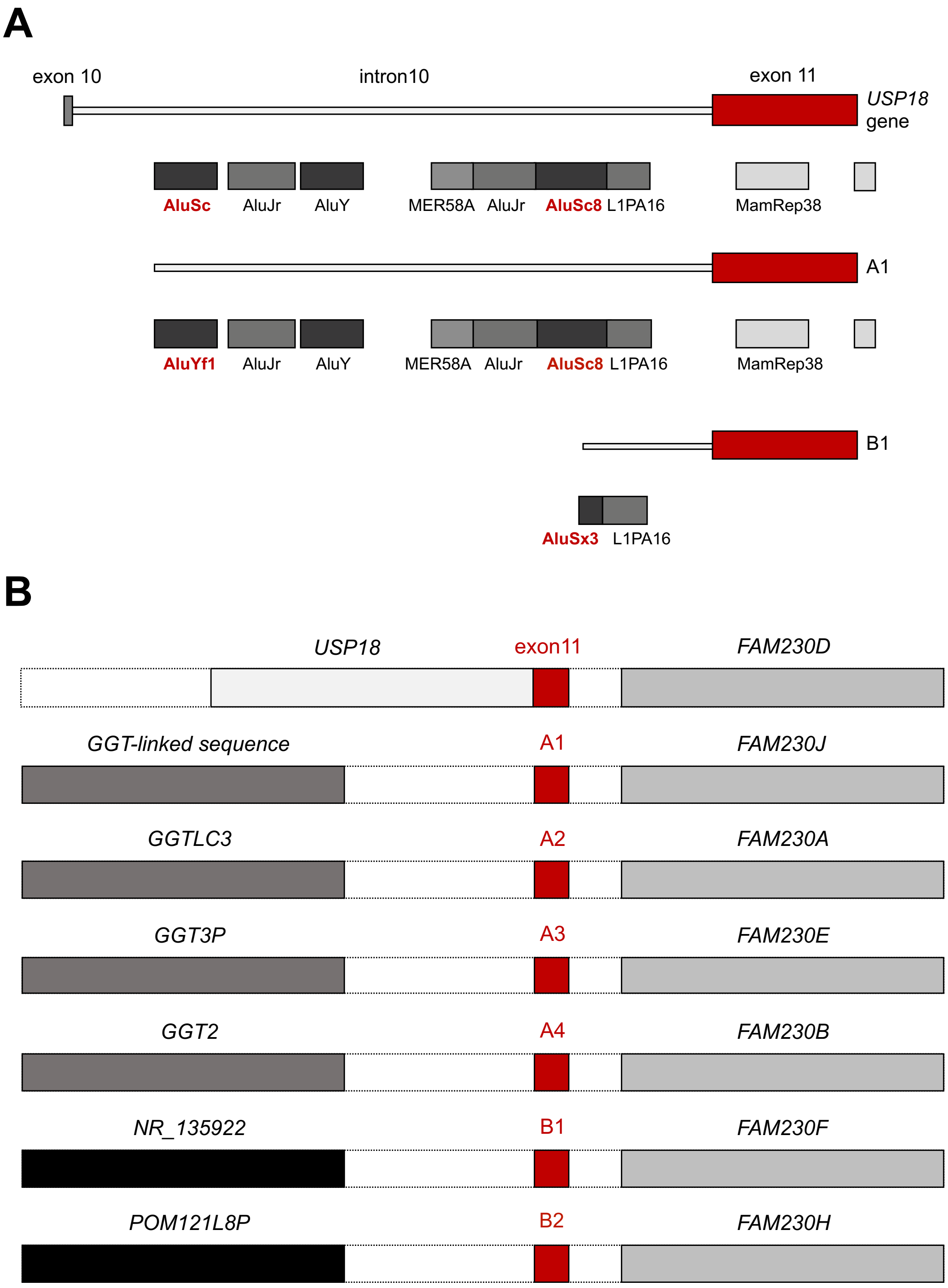
A
exon 10
intron10
exon 11
USP18 gene
AluSc
AluJr
AluY
MER58A
AluJr
AluSc8
L1PA16
MamRep38
A1
AluYf1
AluJr
AluY
MER58A
AluJr
AluSc8
L1PA16
MamRep38
B1
AluSx3
L1PA16
B
USP18
exon11
FAM230D
GGT-linked sequence
A1
FAM230J
GGTLC3
A2
FAM230A
GGT3P
A3
FAM230E
GGT2
A4
FAM230B
NR_135922
B1
FAM230F
POM121L8P
B2
FAM230H

Supplementary Figure S9. ***USP18*** **intron 10-exon 11 copies contain** ***Alu*** **elements at their breakpoints and are part of repeated gene blocks.**

(A) Repeated elements present in the A and B copies (A1 and B1 are shown as representatives). The *Alu* sequences at the breakpoint of the A and B copies are shown in red. Exons are shown as boxes, intron as thick lines. Sequences identical to *USP18* exon 11 are in red.

(B) *USP18* exon 11, A copies and B copies are shown in red. *FAM230*-linked sequences/genes are found downstream of A and B copies. GGT-linked sequences/genes are shown upstream of A copies. POM121-related sequences/genes (*POM121L8P* and *NR_135922*) are found upstream of the B copies. Genes/sequences are shown with their genomic orientation (> for + strand, < for – strand).

Supplementary sequences

A) Human USP18 3'UTR

The cDNA sequence of USP18 exon11 is shown in red. The 43 coding nt are in bold, the stop codon (TAA) is underlined, the rest of exon11 represents USP18 3'UTR. This sequence was downloaded from UCSC genome browser.

GCAGGAAACTGCATATCTTCTGGTTTACATGAAGATGGAGTGCTAATGGAAATGCCCAAAACCTTCAGA
GATTGACACGCTGTCATTTTCCATTTCCGTTCCTGGATCTACGGAGTCTTCTAAGAGATTTTGCAATGAGGAG
AAGCATTGTTTTCAAACTATATAACTGAGCCTTATTTATAATTAGGGATATTATCAAAATATGTAACCATGAG
GCCCCTCAGGTCCTGATCAGTCAGAATGGATGCTTTCACCAGCAGACCCGGCCATGTGGCTGCTCGGTCCTG
GGTGCTCGCTGCTGTGCAAGACATTAGCCCTTTAGTTATGAGCCTGTGGGAACTTCAGGGGTTCCCAGTGGG
GAGAGCAGTGGCAGTGGGAGGCATCTGGGGGCCAAAGGTCAGTGGCAGGGGGTATTTCAGTATTATACAAC
TGCTGTGACCAGACTTGTATACTGGCTGAATATCAGTGCTGTTTGTAATTTTTCACTTTGAGAACCAACATTA
ATTCCATATGAATCAAGTGTTTTGTAACTGCTATTCATTTATTCAGCAAATATTTATTGATCATCTCTTCTCCA
TAAGATAGTGTGATAAACACAGTCATGAATAAAGTTATTTTCCACAAAA

B) FAM247A, FAM247C, FAM247D

These transcripts have nearly identical sequence. The following cDNA sequence was obtained by sequencing two overlapping PCR products: the first obtained with A_FW1 primer and AUAP_RV and the second with the A_FW2 primer and the 3'UTR_RV primer (**Figure 5A**). Of note, the genes of FAM247A, C and D are annotated in the UCSC database as LOC105372935, LOC105377182 and LOC105372942 respectively, and the corresponding annotations for transcripts are XR_938017.2, XR_951239.1 and XR_951230.1.

TGAGGCATGAGTTTGGCCACTCCGTAGTGTGCACTTGGTGAGGGCAGCAGCTCGCCACAGCTGCCAGCCATC
TGTCCATTCACCCATCTGTCCATCTGGCAGCCCGCTGTTCAGACCTGTCTGTCTGTCCGCCCATCTGTAAGCC
CATCTCTGTCCCATTGTCTATCTGACCATCTTTCTCTTACTGTCCTCTTTGTCTAGCTATCTGGCCTGTCTGTCG
ATCCATCTTCGTGTCTGTCTTCAGCCCCCACCTGTTTGTCCATCTGTCCAATTACCTGTGAGTCTATCTATGCA
TCTTCTTGTCCATTCATCTGCCCACCCATCTGTCCCTCCATCTGCCCACCGGCCTCCCCTCTCCTTCTGGGCCG
CAGAGCCATGGCCCAGGACTACGGAGCCATGGGTGACCTGGTCCTGCTGGGGCTGGGGCTGGGGCTGGCGC
TGGCTGTCATTGTGCTGGCTGTGGTCCTCTCTCGACACCAGGCCCCATTTGACCCCCGGCCTTTGCCCACACC
GCTGTTGCTGCTGACTCCAAGGTCTTCTCAAATATTGTACG**GCAGGAAACTGCATATCTTCTGGTTTACAT**
GAAGATGGAGTGCTAATGGAAATGCCCAAAACCTTCAGAGATTGACACGCTGTCATTTTCCATTTCCGTTCC
TGGATCTACGGAGTCTTCTAAGAGATTTTGCAATGAGGAGAAGCATTGTTTTCAAACTATATAACTGAGCCTT
ATTTATAATTAGGGATATTATCAAAATATGTAACCATGAGGCCCCTCAGGTCCTGATCAGTCAGAATGGATG
CTTTCACCAGCAGACCCGGCCATGTGGCTGCTCGGTCCTGGGTGCTCGCTGCTGTGCGAGACATTAGCCCTTT
AGTTATGAGCCTGTGGGAACTTCAGGGGTTCCCAGTGGGGAGAGCAGTGGCAGTGGGAGGCATCTGGGGGC
CAAAGGTCAGTGGCGGGGGGTATTTCAGTATTATACAACTGCTGTGACCAGACTTGTATACTGGCTGAATAT
CAGTGCTGTTTGTAATTTTTCACTTTGAGAACCAACATTAATTCCATATGAATCAAGTGTTTTGTAACTGCTAT
TCATTTATTCAGCAAATATTTATTGATCATCTCTTCTCCATAAGATAGTGTGATAAACACAGTCATGAATAAA
GTTATTTTCCACAAAA

C) Partial *linc-UR-B1*

Linc-UR-B1 is a fusion of *NR_135922*, the genomic sequence between the annotated NR_135922 and *TCONS_00029754*, and *TCONS_00029754*. This partial sequence was obtained by sequencing two overlapping PCR products: the first obtained with B_FW1

primer and AUAP_RV (**Figure 5B**) and the second with the B_FW6 primer (pairing to the exon6 of *NR_135922*) and the 3'UTR_RV primer (**Figure 5C**). The different parts of *linc-UR-B1* cDNA sequence are shown in different colours, depending on their origin: partial exon6 of *NR_135922* in grey (nt in white), intergenic region in white (nt in grey), the annotated exon1 of *TCONS_00029754* in black and the *USP18* exon11 in red. The ATG formed by the junction of *USP18* exon11 and the upstream exon is underlined, as well as the stop codon (TAA).

TTTCCAGGATCAGACTAACGGCTAAAGAGACTGATGCATCAACACCAGGCAGAGAATAAAGCAGATTT
TTTTTGTTTTGTTTTGGAGAGCCTCTAGGAACTTGAAAAATACATATGCCACACTCTTAAGACCCGGTG
GTTCTTAATCAGGGATGTTCATTAAAATGCTGGAAAACTCTAAAGATTTCCAGGTCCCATCCAAGGAG
ATTTTGCTTCTGATTGACTGGCTAGTGGCCTGGCCATTGGTATTTTGAAAAATCCCTCCAAGTGATTCT
TTTACATCCCAGCTAGAAAACCCTAAATTAAAGGTGAAAAACCAGACACCAAGTGGCATTTAAATAAA
TGTCAACTTTAACTCCACAAAGCATCTGGTTGCATGTGGACAGAAAGAGAAGGAAAGAGGGCCCTATA
TCTGGATAACTTGGAAATGTGCTCCCCCTAGCAAGATATCTACCAAAATTAAAACCATATTTGAGGATG
CTGGCACTGTGAGCAATATATAAATGATGCATGTAACATCATTTAATATGATCTTATTTTTAAAAATGA
GTAGAGTGGTGTTTTCTAGCTGTTAGTGTTTCCAAATATCAATGTAGAAATTAGCCTTCTGCAGCTGCA
GAGGCAATTCAGTTTGCAGCTTGCTTGCATGTGGCCTAGAGCCACCCAGCCTGATATGTACTAATTTT
TTATGTTTAACTTGCCAGAGTAGAAACTCAGTTTCTGGGCGAGGCATAGTGGCTCATGCCTGTAATCC
CAGAACTTTGGGAGGCCAAGGCAAGCAGATCACAAGGTCGGGAGTTCAAGACCAGCCTGGCTGACAT
AGTGAAACCCTATCTTTGCTAAAAATACAAAAATTAGCCCGGCATGGTGGCAGGCACCTGTAGTCCCA
GCTACTTGGGAGGCTGAGGCAGGAGAATCACTTGAACCTGGGAGGCAGAGGTTGTGGTGAACTGAGA
TCATGCCCCTGCACTCCAGCCTGGGCAACAGAGTGAGACTCCATCTCAAAAAAAAAAAAAAAAAGAAA
CTCAGTTTCTGGTTACATCTGATCTTTATTTTTTATATATCATCTAAGCTATAAAGTTATATTCCCTATTTGTGA
TCTTAAAAGAAGGACTCCAGGAAAGTGTTCAAATATTCATATATCTAAACTGGAACATATGTTTATATTTTTA
AAAGTAGCCTGAGAGGTTGGCAACTAAAGTCATATGTTGAATGATCATTTCTCAAGAGTTTCATTTTATGGTC
TTTCTCTTGTTCTGTAAAATGTGGGCATGGATAGATATAAAGTGCCTGGTGTCCATGCTTTTGTGAAATCCCT
TCCTCTTCCATGTGAATGGGACCTGTGACTTTCTTCTAACCCAGAGAACACAGCAAAAATGATGTGATTTATC
TGAGTCCATTGATGA**CATTGATTACGACTTCCCTTCACCACATTATTTAGGACTGCGTCGTAGGAGACT**
GGGACACATATCCACTTTGCTGGCTTGATGAAGTAAACTGCTAAGTTGAGGAAGCCCACATGGCAAGG
AACTGTGGGCAGCCTTCAGCCAACAGGCAGCAAAAAGCTGAGCTCCTCGGAGCTACAGCCTCAAGGA
AGTTACTTCTGCTAACAACCTGAAAAAGCTTGGAAGCTAGTTTCTCTCTAGTGGAATTTTTAGGGAAGA
GCATGGCCCAGGCAAGATAAATAATGCAGGAAACTGCATATCTTCTGGTTTACATGAAGATGGAGTGC
TAATGGAAATGCCCAAAACCTTCAGAGATTGACACGCTGTCATTTTCCATTTCCATTCCTGGATCTACGGAGT
CTTCTAAGAGATTTTGCAATGAGGAGAAGCATTGTTTTCAAACTATATAACTGAGCCTTATTTATAATTAGGG
ATATTATCAAAATATGTAACCATGAGGCCCCTCAGGTCCTGATCAGTCAGAATGGATGCTTTCACCAGCAGA
CCCGGCCATGTGGCTGCTCGGTCCTGGGTGCTCGCTGCTGTGCGAGACATTAGCCCTTTAGTTATGAGCCTGT
GGGAACTTCAGGGGTTCCCAGTGGGGAGAGCAGTGGCAGTGGGAGGCATCTGGGGGCCAAAGGTCAGTGGC
AGGGGGTACTTCAGTATTATACAACTGCTGTGACCAGACTTGTATACTGGCCGAATATCAGTGCTGTTTGTAA
TTTTTCACTTTGAGAACCAACATTAATTCCATATGAATCAAGTGTTTTGTAACTGCTATTCATTTATTCAGCAA
ATATTTATTGATCATCTCTTCTCCATAAGATAGTGTGATAAACACAGTCATGAATAAAGTTATTTTCCACAAA
A
